# Comparing Approaches to Teaching Patients How to Use an App-Based Home Spirometer: Randomized Controlled Trial

**DOI:** 10.2196/74125

**Published:** 2025-08-14

**Authors:** Caitlin Morgan, Daniel Higbee, Catherine Dixon, Emma Buckroyd, Huzaifa Adamali, Shaney Barratt, Rahul Shrimanker, Catherine Hyams, Paul White, James William Dodd

**Affiliations:** 1 Academic Respiratory Unit, Southmead Hospital, Southmead Road, Bristol, BS10 5NB, United Kingdom; 2 Respiratory Department, Southmead Hospital, North Bristol NHS (National Health Service) Trust, Bristol, United Kingdom; 3 College of Arts, Technology and Environment, University of the West of England, Bristol, United Kingdom

**Keywords:** home spirometry, disease monitoring, medical education, mobile phone, access to healthcare, remote monitoring

## Abstract

**Background:**

Bluetooth-enabled, app-based home spirometry has been validated for use in the diagnosis and monitoring of respiratory disease. Remote teaching (virtual or self-directed) of patients on how to use an app-based home spirometry offers the opportunity to deliver diagnostics safely and at scale. However, the most appropriate method of teaching home spirometry to patients is unknown.

**Objective:**

The aim of this pragmatic study undertaken during the COVID-19 pandemic was to determine whether virtual or self-directed teaching were valid methods for deploying home spirometry to patients referred for outpatient lung physiology testing.

**Methods:**

REACH-SPIRO is a single-center, unblinded, randomized controlled trial of adults referred for spirometry. Participants were randomized (1:1:1) to be taught to use a Bluetooth, app-based spirometer either (A) face-to-face, (B) virtually (live video conferencing), or (C) self-directed. Forced vital capacity (FVC) and forced expiratory volume in 1 second (FEV_1_) were recorded. Home spirometry (Spirobank Smart Spirometer) readings for each training modality were compared with gold standard hospital measurements (Vyaire Medical) using Bland-Altman Limits of Agreement (LoA). Patients' feedback questionnaires on acceptability and adherence were also collected, and common themes were described using content analysis.

**Results:**

A total of 106 participants were randomized. Bland-Altman analysis between hospital and home FEV_1_ measurements for group A showed a mean difference of 0.108L, LoA −0.331L to 0.548L, group B 0.152L, LoA −0.358L to 0.661L, and group C 0.153L, LoA −0.358L to 0.661L. FVC measurements in group A showed a mean difference of 0.123L, LoA −0.402L to 0.648L, group B 0.249L, LoA −0.297L to 0.795L, and group C 0.340L, LoA −0.556L to 1.235L. There was no significant difference between randomized arms for either mean FEV_1_ (*P*=.41) or FVC (*P*=.84). Patient feedback was similar across all groups, with more positive feedback for face-to-face and virtual teaching methods.

**Conclusions:**

There is no meaningful difference in spirometry measurement between patients taught to use a home spirometer remotely (virtual or self-directed) versus traditional face-to-face treatment. Patients’ feedback was favorable to all three methods of teaching. This study supports the use of either virtual or self-directed teaching of home spirometry for the monitoring of respiratory disease. In doing so, we can improve access to spirometry in communities facing barriers to health care and clinically vulnerable populations.

## Introduction

Bluetooth-enabled handheld spirometry devices have emerged as valuable tools for remote lung function monitoring, particularly for respiratory conditions such as interstitial lung disease, airway disease, and cystic fibrosis [1-3], but also other specialties, including cardiology and neurology. It requires participants to conduct forced breathing maneuvers, which measure volume and flow of expired air. The gold standard, hospital spirometry, involves specialist equipment and training to perform and interpret.

Home spirometry devices have been used effectively in the prompt management of exacerbations (flares of disease), early detection of disease decline, and preventing cross-infection in vulnerable patient groups [1-3]. During the COVID-19 pandemic, spirometry was considered an aerosol-generating procedure posing an infection risk requiring considerable adaptations, including preclinic screening and full personal protective equipment, which ultimately reduced testing capacity and placed a significant burden on health systems. Home spirometry offers a solution in terms of increasing the capacity of physiology services, but the most appropriate method of teaching home spirometry to patients is unknown.

The validity of different home spirometry devices has been assessed in terms of their use diagnostically and in monitoring for disease progression, with some adopted into the clinical setting. It is widely recognized that home spirometry measurements, including forced expiratory volume in 1 second (FEV_1_) and forced vital capacity (FVC), are often lower when compared to hospital spirometry, but the reliability of devices has been inferred by the repeatability shown with recurrent measurements [2-7].

Good quality home and hospital spirometry measurements are based on the Association for Respiratory Technology and Physiology (ARTP) guidelines and imply good supervision and understanding of the technique. Reduced supervision by a skilled physiologist, differences in teaching techniques, and differences in equipment are the key barriers to producing good quality home spirometry described in the literature [6,8,9]. Qualitative analysis of how patients view home spirometry has also highlighted the importance of bespoke use of devices according to patients' needs, so as not to risk psychological well-being [10].

This randomized controlled trial (RCT) evaluates 3 methods of teaching patients to use the MIR (Medical International Research) Spirobank Smart spirometer and assesses clinical and statistical differences in spirometry performance. The randomized groups included face-to-face teaching, virtual teaching, and self-directed learning. Additionally, patient feedback was collected to identify barriers to home spirometry adherence and assess the potential impact on the quality of measurements. This study aims to provide evidence as to whether remote teaching (self-directed or virtual) is an acceptable method by which to deploy home spirometry in the community. By doing so, this study hopes to enhance access to diagnostics, reduce health care burdens, and contribute to sustainable health care practices. Some of the results of this study have been reported previously in the form of an abstract [11].

## Methods

### Aim

We aim to determine if there is a difference between virtual, self-directed, and face-to-face teaching of home spirometry and gather patients’ feedback on willingness to perform quality home spirometry with the MIR Spirobank Smart spirometer.

### Design

**Figure 1.**
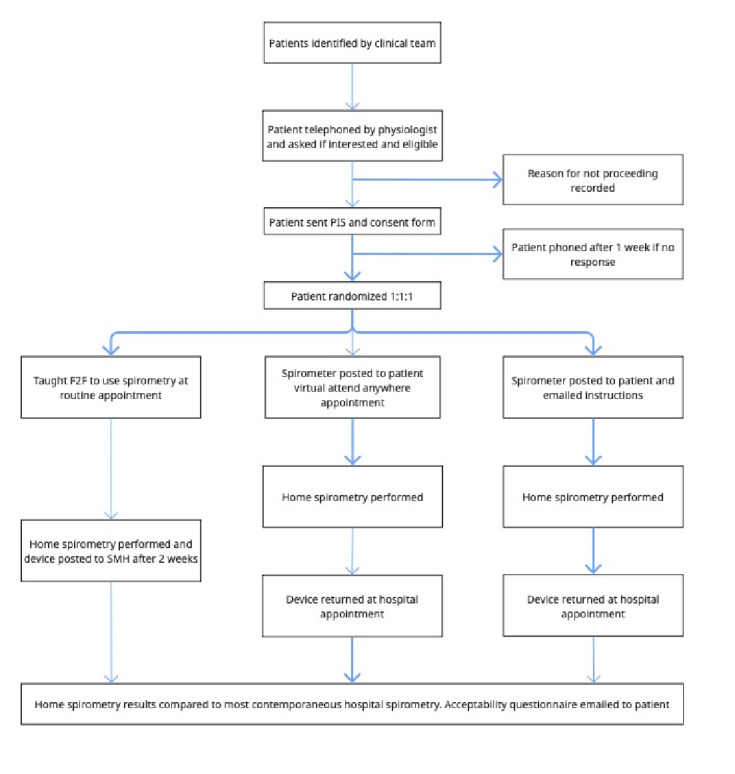
REACH-SPIRO randomized controlled trial design, Bristol, UK: recruitment from July 2021 to July 2022. Randomization groups shown 1:1:1. F2F: face-to-face; PIS: patient information sheet; SMH: Southmead Hospital.

This pragmatic, single-center, unblinded RCT compared virtual, self-directed, and face-to-face teaching of home spirometry on the Spirobank Smart spirometer in respiratory patients who required spirometry as part of their standard care for diagnosis or monitoring of disease at a central Bristol Hospital, UK (ISRCTN:18299685, Research Ethics Committee [REC] 21/NI/0063). It was designed, set up, and launched during the social distancing rules established in the SARS-CoV-2 pandemic with recruitment from July 2021 to May 2022. Participants were randomized to 1 of 3 groups: (A) face-to-face teaching (control), (B) virtual teaching, and (C) self-directed learning (Figure 1). Participants were asked to submit 1 or more home spirometry readings during this study’s period. Statistical analysis was unblinded. The differences in FEV_1_ and FVC between hospital (Vyaire Medical) and home spirometry were compared, and the differences in values between virtual, self-directed, and face-to-face teaching were also compared. Patient feedback was evaluated using feedback questionnaires. No changes were made to the methodology after trial commencement.

### Participants

We included adult patients (aged ≥18 years) referred for hospital spirometry by primary or secondary care with respiratory symptoms. Participants were eligible if they were able to give written informed consent and they owned a smartphone or tablet with an internet connection that they were willing to use for this study. We excluded those with a contraindication to spirometry as per ARTP guidance [12].

### Outcome Measures

Primary outcome: a comparison of FEV_1_ and FVC measurements between face-to-face, virtual, and self-directed teaching of home spirometry using matched in-hospital spirometry measurements.

Secondary outcomes: understand and assess factors that affect patient willingness, adherence, and quality of home spirometry.

### Recruitment and Randomization

All patients under the care of the North Bristol NHS (National Health Service) Trust (NBT) Respiratory service were eligible for recruitment. Patients had to be aged older than 18 years, require spirometry as part of their routine clinical care, have a smartphone and internet connection, and be able to give written informed consent. Patients were identified by a member of their clinical team as requiring spirometry for the diagnosis or monitoring of a respiratory disease. They were approached within 3 weeks of their hospital referral via telephone by a member of the physiology team and screened for eligibility. If provided, reasons for declining to participate were recorded. Consented patients were randomized using a secure web-based system (REDCap [Research Electronic Data Capture; Vanderbilt University]). Participants were randomized in a 1:1:1 ratio to either face-to-face teaching (control arm), virtual teaching, or self-directed learning. It was not practical to use blind interventions in this study. Randomization was not stratified.

### After Randomization

#### (A) Control Arm (Face-to-Face Teaching)

Patients allocated to the control arm attended the hospital for their routine laboratory-based hospital spirometry. They had a face-to-face teaching session with a physiologist who helped download the app to the patient’s phone, set up the Spirobank Smart Spirometer, and perform spirometry using the equipment. The patient practiced the maneuvers with the physiologist. They then performed spirometry at home using the Spirobank Smart spirometer 1 or more times. This was supplemented by the NBT Home Spirometry Patient Information Leaflet and access to the Spirobank Smart spirometer patient information video produced by NBT and available on the NBT website.

#### (B) Intervention Arm 1 (Virtual Remote Teaching: National Health Service Attend Anywhere)

Participants allocated to the first intervention arm were sent the home spirometer and information leaflet by post. This was supplemented by the instructional video. Participants then had a virtual appointment with a member of the respiratory physiology team. At this virtual appointment, they were taught how to use the spirometer and app. They performed spirometry during the virtual appointment and then at home 1 or more times. Patients attended their routine in-hospital spirometry appointment after they had submitted measurements using the home device.

#### (C) Intervention Arm 2 (Self-Directed Learning)

Participants allocated to the second intervention arm were sent the spirometer and information leaflet by post. This was supplemented by the instructional video. They performed spirometry at home 1 or more times. Patients attended their routine in-hospital spirometry appointment after they had submitted measurements using the home device.

#### All Randomized Arms

Participants were asked to record their spirometry at least once, but as often as they liked or felt was clinically indicated. The most contemporaneous home and hospital measurements were used in the final analysis to assess diagnostic utility. All spirometers were calibrated before being issued to each participant, and spirometers were used according to the guidelines. All patients were invited by email to provide feedback questionnaires after returning the home equipment. A 1‐10 Likert scale was used to answer questions on the ease of use of the spirometer, the app, and whether they would want to continue using the home spirometry device. Free text boxes were also provided for feedback on the “challenges to performing home spirometry” and “feedback on the teaching method used.” A question on whether harm was caused as a result of home spirometry was also included in the questionnaire (see Multimedia Appendix 1). The questionnaire is not a validated tool for analysis in this setting and was created for the purposes of this study based on common barriers to home spirometry in the literature.

#### Sample Size

This is a pragmatic trial based on the number of patients referred for spirometry across this study’s period. Given the time-sensitive nature of improving diagnostics and monitoring strategies for vulnerable patient groups during the COVID-19 pandemic and the long wait times of current spirometry services, a formal power calculation was not conducted.

#### Acceptability

Acceptable spirometry measurements are defined by European Respiratory Society and American Thoracic Society criteria as summarized by the most recent ARTP statement on pulmonary function testing [13,14]. Acceptability was assessed by trained physiologists.

### Statistical Analysis

#### Primary Outcome

The differences in FEV_1_ and FVC between hospital (Vyaire Medical) and home spirometry in the three arms were initially assessed using independent samples *t* tests. Within each arm, limits of agreement between hospital (gold standard) and home spirometry were assessed using Bland-Altman methodology (set to 2 SDs from the mean), with agreement bias between home and hospital measures quantified using 95% CIs. Per-protocol analysis, including only those who had produced acceptable hospital spirometry readings (per European Respiratory Society and American Thoracic Society criteria), was also performed at each stage to reduce bias introduced by unacceptable readings (available in Table S1 in Multimedia Appendix 1).

#### Secondary Objectives

Of those who submitted a questionnaire after completing this study, median scores and IQR were calculated for questions 1‐3 of the feedback questionnaire, and content analysis was used to analyze the responses in the white boxes. The results of the qualitative analysis were combined with frequency statistics of missing data and “unacceptable results” (felt to reflect quality of home spirometry readings and therefore quality of teaching) to assess willingness, quality, and adherence to home spirometry.

### Ethical Considerations

This study was fully approved by the Health Research Authority (HRA) Research Ethics Committee Northern Ireland (REC reference 21/NI/0063). The full study protocol has been registered with the ISRCTN registry (ISRCTN18299685). All participants were recruited with informed written consent by means of participant-dated signature by a member of the research team with GCP training. Informed written consent was obtained after the participant reviewed the patient information sheet detailing the exact nature of this study and any risks involved in taking part. Data used in the analysis, both quantitative and qualitative, were anonymized. Participants were not compensated for their time as this study did not involve any extra visits outside of standard clinical appointments.

## Results

### Overview

Between July 13, 2021, and May 26, 2022, a total of 263 patients were screened and 106 were randomized. Principal reasons for exclusion from the study were patient choice not to be involved in this study (24/157, 19%), and an inability to use the technology or a home spirometer (23/157, 15%). The last patient completed this study on July 11, 2022. As per the study protocol, the study was open for 1 year. Baseline characteristics are described in Table 1.

**Table 1.** REACH-SPIRO randomized controlled trial participant flow diagram, Bristol, UK: recruitment from July 2021 to July 2022. Baseline demographic and clinical characteristics for each randomized group by age, gender, previous spirometry experience, and background general practitioner diagnosed respiratory condition.

Characteristics	Face-to-face teaching (n=34)	Virtual teaching (n=36)	Self-directed teaching (n=36)
Age (years)	57	53	58
Minimum-maximum (years)	30-74	29-73	34-82
Female sex, n (%)	19 (56)	21 (58)	24 (67)
Previous spirometry experience ever, n (%)	12 (35)	7 (19)	10 (28)
Hospital spirometry	8 (24)	6 (17)	6 (17)
GP^a^ spirometry	4 (12)	1 (3)	4 (12)
Any respiratory diagnosis, n (%)	16 (47)	13 (36)	13 (36)
Asthma	9 (26)	8 (22)	9 (26)
COPD^b^	2 (6)	1 (3)	3 (8)
Mixed airways	2 (6)	0 (0)	0 (0)
ILD^c^	3 (9)	2 (6)	1 (3)
Other	0 (0)	2 (6)	0 (0)

a GP: general practitioner.

b COPD: chronic obstructive pulmonary disease.

c ILD: interstitial lung disease.

### Data Completeness

**Figure 2.**
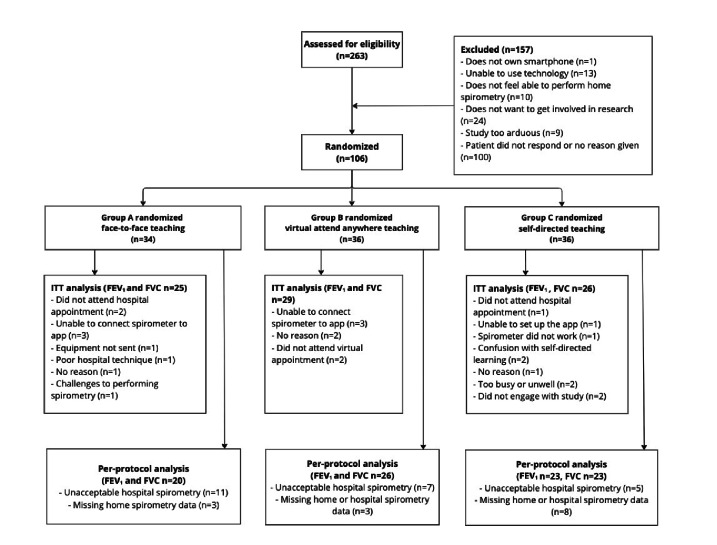
REACH-SPIRO randomized controlled trial participant flow diagram, Bristol, UK: recruitment from July 2021 to July 2022. Randomization groups showed 1:1:1 with final intention-to-treat and per-protocol recruitment for analysis. Exclusion and drop-out reasons were documented. FEV_1_: forced expiratory volume in 1 second; FVC: forced vital capacity; ITT: intention-to-treat.

Missing home and hospital measurements for the 2 metrics analyzed are outlined in Figure 2. A total of 100% (85/85) of those who provided both hospital and home spirometry measurements provided feedback.

### Clinical Outcomes

#### Baseline Demographics

As shown in Table 1, the mean participant age was 56 years, with 60% female and 95% of White ethnicity. Preexisting diagnoses were recorded based on general practitioner records. Asthma was the most common in all groups (n=9, 26% in groups A and C and n=8, 22% in group B). Previous spirometry experience was based on whether participants had ever performed hospital or general practitioner–based spirometry. This varied across groups, with the highest in group A (n=12, 35%).

#### Primary Outcome: Difference in FEV_1_ and FVC Measurements Between Teaching Methods

**Figure 3.**
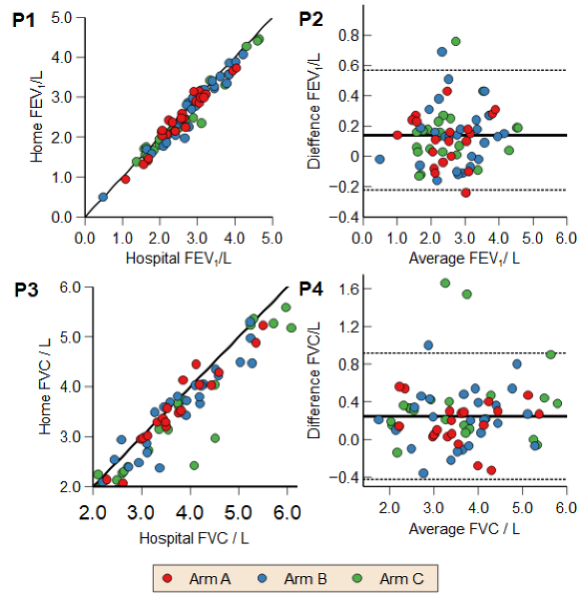
REACH-SPIRO randomized controlled trial, Bristol, UK: recruitment from July 2021 to July 2022. Comparison of teaching methods by randomized arm for FEV_1_ and FVC: A=face-to-face, B=virtual, and C=self-directed; P1 FEV_1_ correlation coefficient scatterplot of all randomized groups. P2 FEV_1_ Bland-Altman plot of all randomized groups. P3 FVC correlation coefficient scatterplot of all randomized groups. P4 FVC Bland-Altman plot of all randomized groups. FEV_1_: forced expiratory volume in 1 second; FVC: forced vital capacity.

The median number of days between hospital and home spirometry measurements was 3 (IQR 1‐9) in group A, 3 (IQR 1‐7) in group B, and 7 (IQR 1‐14) in group C. The difference in means between home and hospital measurements does not significantly vary between randomized arms in FEV_1_ (*F*_2, 76_=2.262, mean squared error=2724.0, *P*=.11) or FVC (*F*_2, 78_=2.595, mean squared error=0.059, *P*=.08). Table 2 and Figure 3 compare FEV_1_ and FVC measurements on the MIR Spirobank device at home versus hospital readings. The limits of agreement for the Bland-Altman plots were set to 2 SD from the mean.

**Table 2.** REACH-SPIRO home spirometry randomized controlled study, Bristol, UK: recruitment from July 2021 to July 2022. Intention-to-treat analysis in all randomized groups. Correlation between home and hospital measurements, mean difference, and limits of agreement with 95% CIs.

Variables	Pearson correlation between home and hospital (95% CI)	*P* value	Agreement bias or mean difference	Bland-Altman limits of agreement (95% CI)
FEV_1_^a^				
Group A (n=25)	0.972 (0.936 to 0.988)	<.001	0.108	-0.218 to 0.435 (-0.331 to 0.548)
Group B (n=29)	0.972 (0.940 to 0.987)	<.001	0.152	-0.232 to 0.536 (-0.358 to 0.661)
Group C (n=26)	0.981 (0.957 to 0.991)	<.001	0.153	-0.217 to 0.523 (-0.342 to 0.649)
Overall (n=80)	0.975 (0.962 to 0.984)	<.001	0.138	-0.221 to 0.498 (-0.291 to 0.568)
FVC^b^				
Group A (n=26)	0.955 (0.902 to 0.980)	<.001	0.123	-0.402 to 0.648 (-0.581 to 0.827)
Group B (n=29)	0.958 (0.912 to 0.980)	<.001	0.249	-0.297 to 0.795 (-0.478 to 0.976)
Group C (n=26)	0.932 (0.853 to 0.969)	<.001	0.340	-0.556 to 1.235 (-0.860 to 1.539)
Overall (n=81)	0.942 (0.911 to 0.962)	<.001	0.238	-0.462 to 0.937 (-0.596 to 1.071)

a FEV_1_: forced expiratory volume in 1 second.

b FVC: forced vital capacity.

#### Difference Between Hospital and Home Spirometry Readings

Home and hospital spirometry were highly correlated, as shown in Table 2 (Pearson correlation) and in P1 and P3, Figure 3. In line with previous studies in the literature, FVC (mean difference 0.238L, *P*<.001, 95% CI 0.160‐0.315) measurements were higher in the hospital compared to the most contemporary home spirometry measurement performed by each participant. This was the case in all teaching groups. FEV_1_ mean difference (0.138L, *P*<.001, 95% CI 0.097‐0.180) was significantly higher in hospital readings but still within ARTP reproducibility criteria of 150 ml. A subanalysis of only those participants who produced acceptable hospital spirometry readings was performed (Table S1 in Multimedia Appendix 1), but no difference was noted in any outcome from the findings of the intention-to-treat analysis.

#### Participant Feedback Questionnaires

Responses to acceptability questions were rated on a Likert scale of 1‐10 (1=hard, 10=easy), and a median with IQR was calculated. In group A, 28 submitted feedback questionnaires. The response to question 1 “How easy did you find performing spirometry at home?” was 9 (IQR 7-9). The median response to question 2 “How easy did you find it to use the app and email your results?” was 8.5 (IQR 5.5-8.5). Question 3: “How happy would you be to continue to use the device to monitor your lung function at home?” showed a median response of 9.5 (IQR 7.5-9.5). A total of 28 participants submitted feedback in group B. Question 1 showed a median response of 10 (IQR 8-10), question 2 a response of 9 (IQR 7-9), and question 3 a response of 10 (IQR 8.5-10). Finally, 29 participants in group C provided feedback. Question 1 had a response of 8 (IQR 3.5-8), question 2 a response of 9 (IQR 4.5-9), and question 3 a response of 9 (IQR 5.5-9).

#### Challenges to Home Spirometry and Feedback on Teaching Methods

Content analysis was conducted on written participant feedback. Common themes are outlined in Table 3.

**Table 3.** REACH-SPIRO home spirometry randomized controlled trial, Bristol, UK: recruitment from July 2021 to July 2022. Content analysis of participant feedback to “challenges to home spirometry” and “feedback on teaching method” with key themes.

Participant challenges and feedback	Group A (n=28), n (%)	Group B (n=28), n (%)	Group C (n=29), n (%)
Challenges to home spirometry			
Reported issues with the app	9 (32)	3 (11)	5 (18)
Connecting to the internet			
Downloading the app			
Emailing spirometry measurements			
Issues with the home spirometer device	0 (0)	1 (3.5)	0 (0)
Reports of not finding “the time” to conduct tests in an informal setting	3 (11)	2 (7)	1 (3.5)
Receiving an alert that the test was “unacceptable”	2 (7)	2 (7)	4 (14)
No problems reported	4 (14)	0 (0)	2 (7)
No feedback given	10 (36)	20 (71)	17 (59)
Feedback on teaching method			
More teaching requested	3 (11)	3 (11)	3 (10)
More info on downloading or navigating the app			
A demonstration video before each test			
Advice on app warnings			
Positive feedback on teaching	9 (32)	9 (32)	5 (17)
Convenient			
Easy to follow or clear			
Good leaflet			
No feedback given	16 (57)	15 (54)	21 (72)
Connectivity issues with virtual teaching	0 (0)	1 (3.5)	0 (0)

The face-to-face group had the most reported challenges with the home spirometer app on their device and struggled to find the time to perform home spirometry outside of the clinical setting. This group also provided the most feedback. Only 1 participant had issues with the spirometer device (group B). The face-to-face and virtual teaching groups had an equal (n=9, 32%) number of participants reporting positive feedback on the teaching methods used. The self-directed group had fewer positive comments on teaching methods (n=5, 17%). Participants reported alerts on the app advising them that their results were “unacceptable.” Participants noted concern in feedback that this meant they had not performed the test correctly. More participants reported issues with “unacceptable” results in the self-directed arm (n=4, 14% vs n=2, 7% in groups A and B versus C).

As shown in Figure 1, missing data and “unacceptable” spirometry readings were recorded in all 3 groups. This was more apparent in the face-to-face and self-directed groups.

#### Harms

One participant in the virtual teaching group (group B) reported that trying to use the app was “stressful and frustrating.” A total of 2 participants in the self-directed group (group C) expressed a desire for physician interpretation of results. No participants reported any side effects from performing home spirometry.

## Discussion

This is the first RCT comparing methods of teaching home spirometry. It has been shown that there is no statistically significant difference between face-to-face, virtual, or self-directed teaching of home spirometry on the MIR Spirobank Smart spirometer. The qualitative analysis of patient feedback did not identify clear differences in usability or participant willingness and adherence to home spirometry depending on which teaching method was used. This trial supports the use of all 3 methods of teaching and therefore, the use of remote teaching (virtual or self-directed) in clinical practice to improve accessibility.

Participant feedback confirmed that technical difficulties with the app occurred across all 3 groups. Concerns were also raised about the app reporting that readings were “unacceptable” without giving a reason why. This led to concern that the test had not been performed correctly. This finding is supported by a recent study looking at virtual teaching of home spirometry in a cystic fibrosis population [3], which found that adherence after virtual teaching was high, but concerns were raised by participants regarding the accuracy of the results provided. Davis et al [3] also commented on the benefits of virtual teaching in allowing for “top-up” sessions to maintain the technique.

This trial has shown that in the outcomes measured (FEV_1_ and FVC) in each randomized group, home spirometry readings were on average lower than those recorded using the in-hospital Vyntus equipment alongside a trained physiologist (138 and 238 ml, respectively). This is consistent with other studies, including a meta-analysis by Anand et al [7], which concluded that unsupervised spirometry is not a suitable replacement for supervised spirometry after assessing the results of 28 separate studies. A 2018 study, which assessed the Spirobank Smart spirometer in an interstitial lung disease cohort, also found an average FEV_1_ mean difference between hospital and home spirometry of 220 ml even when participants were trained to produce acceptable readings before independent use at home [1]. Similarly, in 2012, the Spirobank Smart spirometer showed an underestimation of FEV_1_ by 5% when compared to clinic readings [8]. Kerwin et al [4] also found home FEV_1_ results were “moderately lower” than clinic readings, despite being an average of weekly measurements, but due to the high correlation between home and hospital measurements, this study concluded that home spirometry remained a reliable method of monitoring disease. Reasons for lower home versus hospital spirometry include the time of day that readings were taken, and also the impact of trained physiologists during in-hospital measurements [1,3,4,8]. Spirometry is a hard technique to implement, and acceptable measurements are challenging even when supported by a trained physiologist in a hospital setting. Therefore, it has been argued that only acceptable measurements should be relied upon clinically [3,9]. Based on this, we performed a subanalysis that included only those participants who performed “acceptable” hospital spirometry measurements, under the assumption that those who were unable to perform acceptable readings under optimum (hospital) conditions were less likely to produce acceptable readings without any supervision (Table S1 in Multimedia Appendix 1). However, this did not show a difference in results with FEV_1_ and FVC, all underestimated in the home measurements by 133 and 259 ml, respectively. We also considered the potential impact of differences in the interval between hospital and home measurements. In our study, this was small (median 3 and 7, IQR 1-14 d) with the largest spread in group C. This short timeframe limits the influence of changes in the clinical state of the patient or the risk of patients forgetting the taught instruction on the spirometry result. However, in those with a large delay, these factors may have impacted the results. Together, our data and others suggest that home spirometry may be best suited to monitoring lung function rather than relying on an off-diagnostic threshold.

This trial has several limitations, including sample size. This was a pragmatic design during the COVID-19 pandemic and may be underpowered. However, it is one of the largest studies looking at home spirometry and one of the only studies comparing virtual or self-directed to face-to-face teaching. Researchers noted that just 9% (14/157) of those approached to take part declined due to a lack of a smartphone or a lack of confidence with the spirometer or app. Our population was young, with an average age of 53‐58 years across the 3 groups, with a wide age range. Unlike other studies, we have not removed outlying results from our Bland-Altman analysis, and as such, we have noted large differences between home and hospital measurements. These limitations do not affect the generalizability of this study, and we were able to conclude that virtual or self-directed teaching of home spirometry was comparable to the gold standard (face-to-face teaching). A further limitation of this study was the lack of standardization for when home measurements were taken. Furthermore, participants were not asked to comment on clinical status during home or hospital measurements. However, in the context of this pragmatic RCT and given that all participants were presenting to clinicians with diagnosed or undiagnosed respiratory symptoms, some variation may be expected. Finally, the feedback questionnaire used in this study was designed for this study and, as such, has not been independently validated. There is no validated questionnaire for use in this setting. The questions were devised using common themes in the literature regarding barriers to home spirometry use.

In conclusion, virtual or self-directed instruction are effective methods for teaching home spirometry, yielding comparable results to face-to-face methods in both the quantitative and qualitative analysis. Like other studies, we have identified differences in home versus hospital readings, which suggest that home spirometry should not replace the gold standard diagnostic spirometry. Spirometry provision in the United Kingdom is unable to meet current demands and is extremely variable in different regions. The REACH-SPIRO trial has contributed evidence to support remote teaching of home spirometry (virtually or self-directed), which could help increase the provision of spirometry in clinical and research settings.

## Supplementary material

10.2196/74125 Multimedia Appendix 1 Per-protocol analysis and REACH-SPIRO RCT Questionnaire.

10.2196/74125 Checklist 1 CONSORT checklist (V2). CONSORT: Consolidated Standards of Reporting Trials.

## ABBREVIATIONS

ARTP: Association for Respiratory Technology and Physiology
FEV_1_: forced expiratory volume in 1 second
FVC: forced vital capacity
LoA: limits of agreement
MIR: Medical International Research
NBT: North Bristol NHS (National Health Service) Trust
RCT: randomized controlled trial
REDCap: Research Electronic Data Capture
