# Supplementary material for: Comparing Approaches to Teaching Patients How to Use an App-Based Home Spirometer: Randomized Controlled Trial
Source: JMIR Form Res. 2025 Aug 14;9:e74125. doi: 10.2196/74125 (PMC12358814; doi:10.2196/74125)

**Table S1.** Per-protocol analysis in all randomised groups.

|  | **Pearson**  **Correlation between home and hospital** | **p** | **Agreement bias/mean difference**  **(95% CI)** | **Bland-Altman Limits of Agreement**  **(95% CI)** |
| --- | --- | --- | --- | --- |
| Group A FEV1  (n = 20) | .976  (.940, .991) | <.001 | 0.108  (0.030, 0.186) | -0.219, 0.435  (-0.347, 0.563) |
| Group B FEV1  (n = 26) | .968  (.929, .986) | <.001 | 0.140  (0.056, 0.224) | -0.267, 0.547  (-0.405, 0.686) |
| Group C FEV1  (n = 23) | .981  (.956, .992) | <.001 | 0.145  (0.063, 0.227) | -0.227, 0.518  (-0.362, 0.652) |
| **All FEV1**  **(n =69)** | **.976**  **(.961, .985)** | **<.001** | **0.133**  **(0.087, 0.178)** | **-0.237, 0.502**  **(-0.314, 0.579)** |
| Group A FVC  (n = 20) | .963  (.902, .985) | <.001 | 0.175  (0.064, 0.287) | -0.291, 0.642  (-0.472, 0.823) |
| Group B FVC  (n = 26) | .945  (.879, .975) | <.001 | 0.230  (0.103, 0.356) | -0.385, 0.843  (-0.593, 1.052) |
| Group C FVC  (n = 23) | .938  (.858, .974) | <.001 | 0.366  (0.173, 0.559) | -0.510, 1.242  (-0.827, 1.559) |
| **All FVC**  **(n = 69)** | **.942**  **(0.908, 0.964)** | **<.001** | **0.259**  **(0.175, 0.343)** | **-0.427, 0.945**  **(-0.569, 1.088)** |
| Group A FEF  (n = 19) | .877  (.704, .952) | <.001 | 0.086  (-0.132, 0.305) | -0.801, 0.974  (-1.146, 1.319) |
| Group B FEF  (n = 26) | .933  (.854, .970) | <.001 | -0.031  (-0.194, 0.133) | -0.825, 0.763  (-1.094, 1.033) |
| Group C FEF  (n = 22) | .899  (.768, .958) | <.001 | -0.159  (-0.359, 0.041) | -1.043, 0.725  (-1.370, 1.053) |
| All  (n = 67) | .909  (.856, .944) | <.001 | -0.040  (-0.146, 0.067) | -0.898, 0.819  (-1.076, 0.996) |
| Group A PEFR  (n = 20) | .944  (.861, .978) | <.001 | 28.90  (10.53, 47.27) | -48.02, 105.83  (-77.92, 135.73) |
| Group B PEFR  (n = 26) | .827  (.647, .920) | <.001 | 58.96  (29.05, 88.87) | -86.19, 204.11  (-135.48, 253.40) |
| Group C PEFR  (n = 22) | .785  (.544, .907) | <.001 | 67.3  (28.1, 106.5) | -106.05, 240.68  (-170.19, 304.83) |
| **All PEFR**  **(n = 68)** | **.834**  **(.743, .894)** | **<.001** | **52.82**  **(35.39, 70.3)** | **-88.38, 194.02**  **(-117.57, 223.22)** |

**Note S1.** Patient feedback questionnaire.


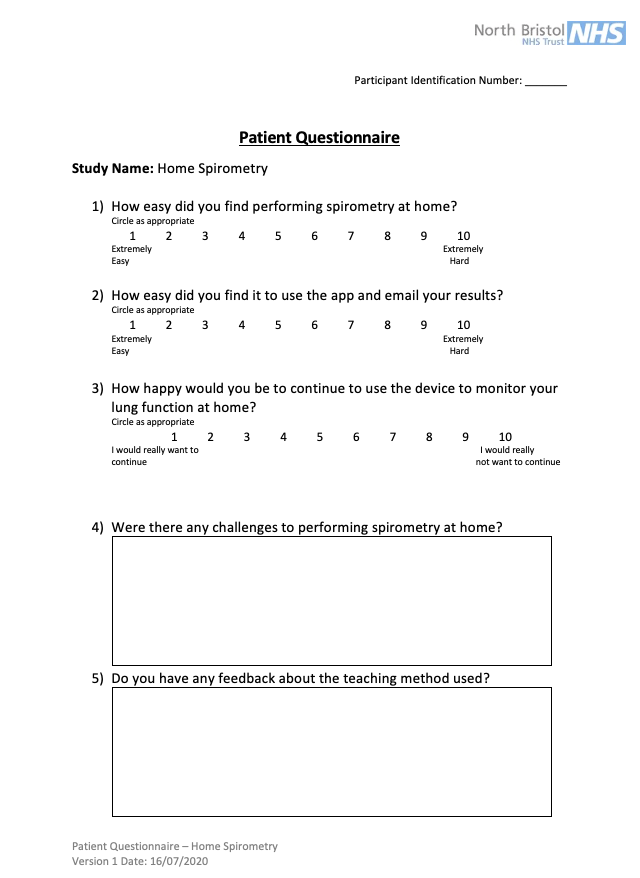


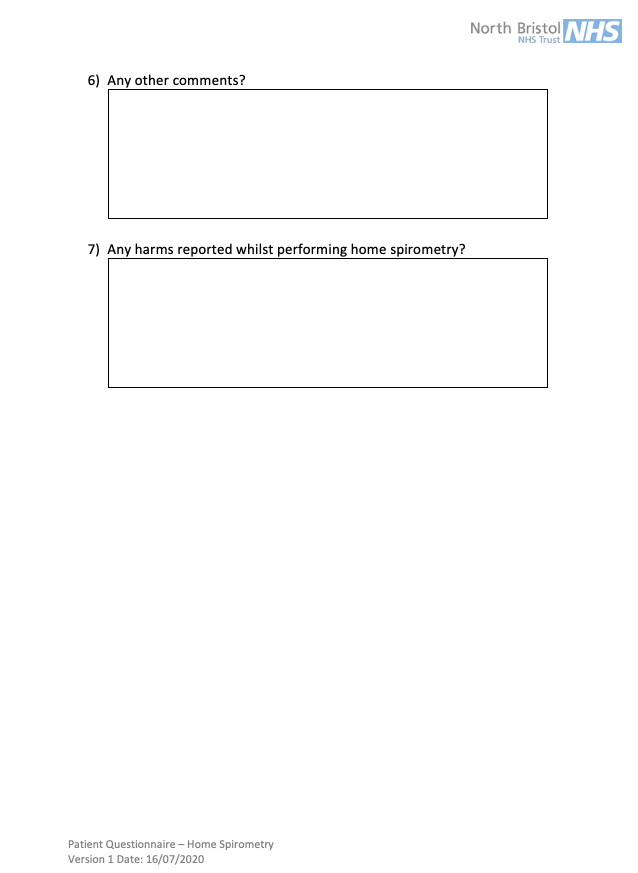

Supplement: Multimedia Appendix 1 [file formative-v9-e74125-s001.docx]
